# SOX10 Expression in Primary Salivary Gland Tumors: A Meta-Analysis

**DOI:** 10.1007/s12105-026-01975-3

**Published:** 2026-10-16

**Authors:** Otto Tahvanainen, Henri Lagerstam, David Kalfert, Ivana Kholová

**Affiliations:** 1 Institute of Clinical Medicine, University of Eastern Finland, Kuopio, Finland; 2 Kuopio University Hospital, Kuopio, Finland; 3 Faculty of Medicine and Health Technology, Tampere University, Arvo Ylpön Katu 34, 33520 Tampere, Finland; 4 Fimlab Laboratories, Pathology, Tampere, Finland; 5 Department of Otorhinolaryngology, Head and Neck Surgery, First Faculty of Medicine, University Hospital Motol and Homolka, Charles University, Prague, Czech Republic

**Keywords:** SOX10, Salivary gland, Meta-analysis, Immunohistochemistry

## Abstract

**Purpose:**

Primary salivary gland tumors form a heterogeneous group with diagnostic challenges. Sex-determining region Y-related homeobox 10 (SOX10) is a transcription factor studied in primary salivary gland tumors for over a decade. To date, no systematic review or meta-analysis has been conducted on its expression in salivary gland tumors.

**Methods:**

A PubMed literature search identified all studies in which SOX10 was evaluated by immunohistochemistry in primary salivary gland tumors. Information on SOX10 positivity, staining pattern, percentage of positive cells, and information on the antibody source and clone were tabulated in Microsoft Excel. The meta-analysis was performed with OpenMeta Analyst. The meta-analysis adheres to PRISMA (Preferred Reporting Items for Systematic reviews and Meta-Analyses) guidelines.

**Results:**

The meta-analysis included 35 studies with 1146 histological and 35 cytological samples immunostained with SOX10. The list of SOX10-positive tumors according to our meta-analysis includes pleomorphic adenoma (pooled meta-analytic estimate of SOX10 positivity 95.8%, 95% CI 91.7–99.9%), polymorphous adenocarcinoma (pooled estimate 97.4%, 95% CI 92.3–102.6%), canalicular adenoma (pooled estimate 98.9%, 95% CI 96.4–101.4%), basal cell adenoma (pooled estimate 91.3%, 95% CI 80.2–102.5%), adenoid cystic carcinoma (pooled estimate 96.6%, 95% CI 94.1–99.2%), myoepithelioma (pooled estimate 75.2%, 95% CI 52.0–98.4%), acinic cell carcinoma (pooled estimate 97.4%, 95% CI 94.1–100.6%), intercalated duct type of intraductal carcinoma (pooled estimate 90.8%, 95% CI 75.9–105.8%), and microsecretory carcinoma (pooled estimate 96.1%, 95% CI 87.2–105.0%). Limited evidence suggests that basal cell adenocarcinoma, epithelial–myoepithelial carcinoma, and myoepithelial carcinoma are also SOX10 positive. SOX10-negative entities include Warthin’s tumor (pooled estimate 2.3%, 95% CI 0–7.0%), apocrine type of intraductal carcinoma (pooled estimate 10.3%, 95% CI − 10.4–31.1%), salivary duct carcinoma (pooled estimate 3.7%, 95% CI − 0.6–8.1%), and oncocytoma (pooled estimate 8.0%, 95% CI − 2.7–18.7%). Polyclonal antibodies and clones EP268, N-20, and A-2 provided similar results. Clone BC34 stained a lower percentage of SOX10-positive tumors.

**Conclusion:**

This meta-analysis lists SOX10-positive and -negative tumors. This demonstrates the necessity of subtype-specific reporting and stating the antibody clone used in immunohistochemical studies.

**Supplementary Information:**

The online version contains supplementary material available at https://doi.org/10.1007/s12105-026-01975-3.

## Introduction

Primary salivary gland tumors are neoplastic diseases that originate from both the major and minor salivary glands. They are a heterogeneous group, with the World Health Organization´s (WHO) Classification of Tumors recognizing 15 benign and 21 malignant tumors [1]. Based on the histological similarities among the entities, the tumors can be classified as basaloid salivary gland tumors, tumors with oncocytic features, and tumors with clear cell features. However, several entities do not belong to any of these groups. This variety poses a diagnostic challenge and makes immunohistochemical studies vital for differentiating salivary gland tumors [2].

Sex-determining region Y (SRY)-related homeobox 10 (SOX10) is a transcription factor involved in the development of the neural crest, cartilage, glial cells, and sex determination. Therefore, it has been used as an immunohistochemical marker for tumors arising from cells with a neural crest origin, such as schwannomas and melanomas [3]. In normal tissues, nuclear SOX10 positivity is encountered in the myoepithelial cells of the mammary, salivary, and bronchial glands. In addition, as noted by Koutlas et al. [4], the positive nuclear staining of SOX10 has been recorded in the acinar, luminal, and abluminal cells of salivary glands.

Although SOX10 positivity in salivary gland tumors has been the subject of constant research for more than a decade, the exact range of SOX10-positive entities remains unclear. For instance, only four tumors are explicitly stated as SOX10 positive in both the WHO Classification of Tumors [1] and the *Diagnostic Immunohistochemistry–Theranostic and Genomic Applications* book [5]: pleomorphic adenoma, myoepithelioma, secretory carcinoma, and intraductal carcinoma. Nevertheless, each book lists another eight [1] and four [5] SOX10-positive tumors, respectively. While entities where SOX10 was not addressed were not necessarily considered SOX10 negative, the difference may demonstrate a differing view of the value of SOX10 for the salivary gland entities. Furthermore, a study with a large selection of entities conducted by Hsieh et al. [6] not only supports the claims of both books but also expands the list of SOX10-positive tumors.

**Fig. 1:**
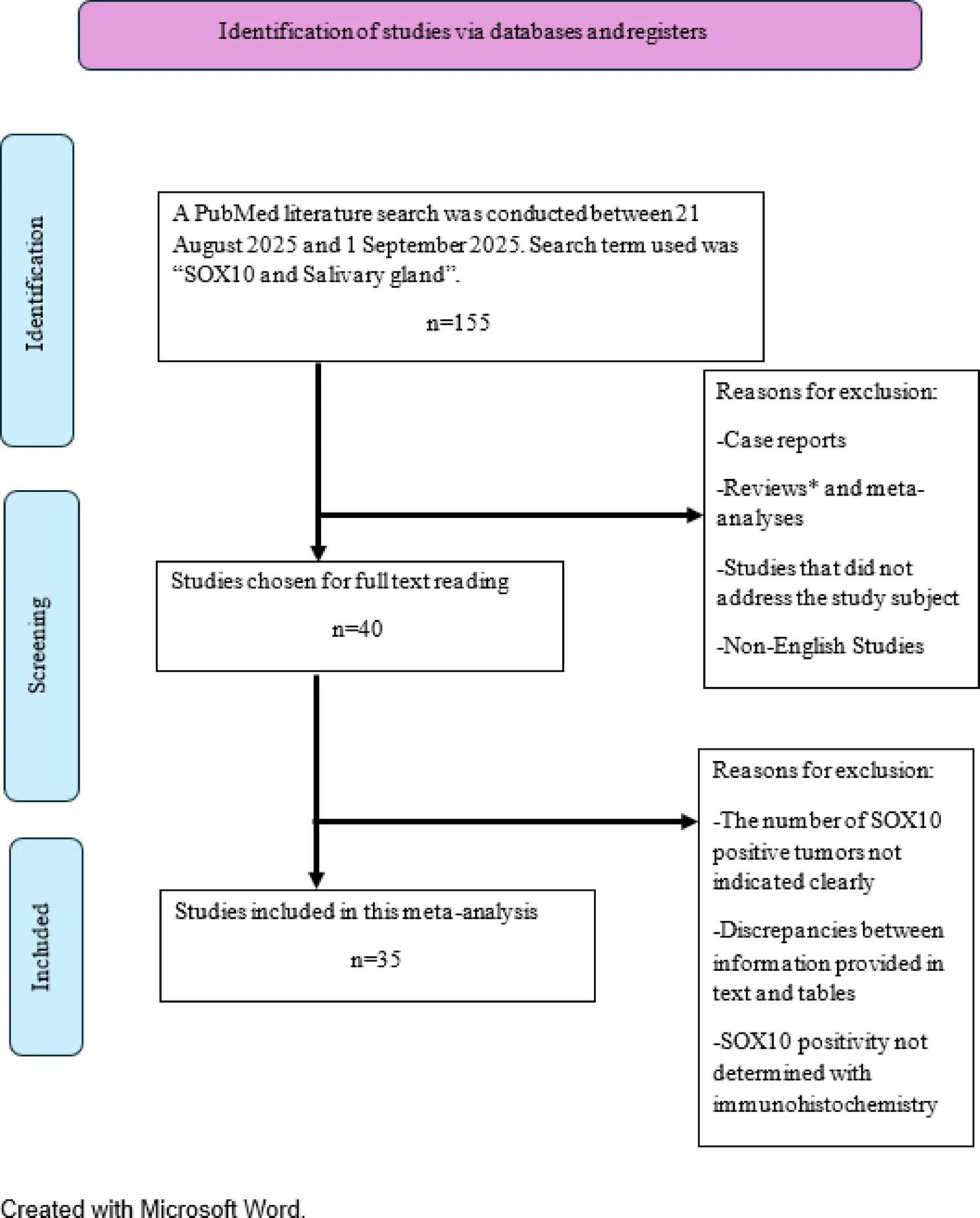
Flow diagram describing the process of identifying studies on SOX10 positivity in salivary gland tumors. *For original studies accompanied by a literature review, only the materials of the original study were considered

Therefore, in this meta-analysis, a literature search found all original studies in which primary salivary gland tumors were immunostained with the SOX10 antibody. The objective of this study was to determine which primary salivary gland tumors are SOX10 positive and how reliable SOX10 is as a diagnostic marker in differential diagnostic work-ups.

## Materials and Methods

### Search Criteria

Originally, the objective of this study was to determine SOX10 expression in the cytological specimens of primary salivary gland tumors. A PubMed literature search was conducted between June 15, 2025, and August 4, 2025. The search keywords used were “SOX10 and salivary gland and (FNA or cytology).” To avoid scenarios in which “salivary gland” was not used in the study, additional search words were used. These included the three major salivary glands (parotid, submandibular, and sublingual glands) and all primary salivary gland tumors described as SOX10 positive in either the WHO Classification of Tumors [1] or *Diagnostic Immunohistochemistry* [5]. The complete list of search terms used is illustrated in Suppl. Table 1. This search yielded 123 articles, one of which fulfilled the criterion for the cytological material studied.

Because the number of cases was insufficient for a meta-analysis, it was decided to expand the goal of this study to include both cytological and histological samples. A PubMed literature search was performed between August 25, 2025, and September 1, 2025, with the search terms “SOX10 and salivary gland.” Since the use of extra search terms in place of “salivary gland” provided no additional benefit when searching for studies on cytological samples, it was concluded that “SOX10 and salivary gland” is sufficient.

### Study Inclusion and Exclusion Criteria

The following criteria were used to include studies in the title and abstract screening: (1) The research includes primary salivary gland tumors, and (2) SOX10 positivity has been studied by immunohistochemistry. Meta-analyses, reviews, case reports, and non-English studies were excluded. However, if a literature review accompanied an original study, the article was not excluded, and only the data from the original study were considered. In a full text reading, studies were excluded if the method used to determine SOX10 positivity was ambiguous or not described. Studies were also excluded if the number of SOX10-positive tumors was not stated or if discrepancies regarding the number of SOX10-positive tumors were detected, for example, between the text and tables (Fig. 1).

### Data Collection

The following data were gathered on a Microsoft Excel sheet (Version 2512, build 16.0.19530.20184, 64-bit): PubMed ID, the first author, year of publication, country of origin, continent, whether the study was institutional, multi-institutional, or international, period of study in years, the type of hospital or laboratory where the study was conducted, and the total number of samples studied. The second Excel sheet contained information on the tumors, themselves: histological and/or cytological diagnosis, tumor grade or subtype, if applicable, the total number of tumors studied and immunostained with SOX10 and the number of SOX10-positive tumors, type of SOX10-positive cells, SOX10 staining pattern, SOX10 staining intensity, percentage of SOX10-positive cells, topography of the tumor and the source, a clone, and the dilution of the SOX10 antibody used. The second Excel sheet was employed for histological samples, and the third Excel sheet was created for cytological samples. The information collected was the same as in the second Excel sheet.

### Meta-Analysis

Statistical analyses were performed using OpenMeta [Analyst] Version 0.24.1. For each tumor entity, the proportion of SOX10-positive cases was pooled across studies using a random-effects model with the DerSimonian–Laird estimator. Quantitative syntheses were performed only for entities evaluated in at least two independent studies. Pooled proportions and corresponding 95% confidence intervals were presented in forest plots. For each tumor entity, two different measures of SOX10 positivity were distinguished. The crude (observed) proportion was calculated as the total number of SOX10-positive cases divided by the total number of evaluated cases across all included studies. In contrast, the pooled proportion represents a meta-analytic estimate obtained by combining study-specific proportions using the random-effects model described above. Therefore, the pooled estimate is not mathematically equivalent to the crude proportion and may differ from the percentage obtained by simply aggregating all positive and negative cases. Throughout the manuscript, the terms “crude/observed proportion” and “pooled meta-analytic estimate” are used to distinguish these measures. Because the confidence intervals are model-based statistical estimates, their limits may occasionally extend slightly beyond the theoretical range of 0–100%. Such values should not be interpreted as biologically possible proportions but as a consequence of the statistical estimation procedure. Between-study heterogeneity was assessed using Cochran’s Q test and quantified using the I^2^ inconsistency statistic. Entities represented by a single study were summarized descriptively. Histological subtypes were analyzed separately when sufficient data were available. The meta-analysis was reported as per PRISMA (Preferred Reporting Items for Systematic reviews and Meta-Analyses) 2020 Guidelines [7].

## Results

### Study Cohort

The literature search yielded 155 articles, of which 40 were selected for a full-text reading based on the inclusion and exclusion criteria. Five studies were excluded after a full-text reading. Of the 35 studies included in the meta-analysis, 14 originated from the USA [8–21], five from Japan [22–26], three from Taiwan [6, 27, 28], three from the Czech Republic [29–31], two from Germany [32, 33], and one each from Brazil [34], Canada [35], Iran [36], Mexico [37], the People’s Republic of China [38], the Republic of Korea [39], Singapore [40], and South Africa [41]. The studies were published between 2013 and 2025. According to the study data periods stated in 22 studies, the series represented at least 343 study years. The number of histological samples across the studies was 1280, of which 1146 were immunostained with SOX10. In total, 35 cytologic samples were also included, all immunostained with SOX10. A flow diagram of the study identification process is presented in Fig. 1, and an overview of the studies is illustrated in Suppl. Table 2.

### Topography of Tumors

All major salivary glands are represented in this meta-analysis, with 226 parotid, 15 submandibular, and two sublingual gland tumors. In total, 165 tumors originated from minor salivary glands (MSG), and the exact location was stated of 98: 25 in the hard palate, 21 in the buccal mucosa, seven in the labial mucosa, six in the palate, five in the upper lip, four in the tongue, three in the lip, three in the soft palate, two in the maxilla, four in the oropharynx, two in the retromolar trigone, and one each in the alveolar ridge, the angle of the mandible, the base of the tongue, the bronchus, the floor of the mouth, the gingiva, the junction of the hard and soft palate, the labial vestibule, the lower lip, the mandible, the nasopharynx, the maxillary sinus, the sphenoid sinus, and the parapharyngeal, the parabronchial, and the pharyngeal compartments. Sixty-seven MSG tumors were in the upper lip, buccal mucosa, and palate. However, the exact number of samples in each location was not stated. Similarly, 39 tumors were in either the parotid or submandibular gland. In addition, 22 tumors were in the parotid, sublingual, and submandibular glands or minor salivary glands in the tongue, the base of the tongue, or the palate. Moreover, 16 tumors were in the parotid gland or lip, accessory parotid, or pharynx MSGs. The location was not reported in 830 (70%) samples.

### Basaloid Salivary Gland Tumors

Basaloid salivary gland tumors include pleomorphic adenoma (PA), basal cell adenoma (BCA), basal cell adenocarcinoma (BCAC), adenoid cystic carcinoma (AdCC), myoepithelial carcinoma, epithelial–myoepithelial carcinoma (EMC), and polymorphous adenocarcinoma (PAC). Table 1 presents the results of this group.

**Table 1:** Summary of SOX10 expression and diagnostic applications in basaloid salivary gland tumors

Tumor type	Studies (n)	SOX10 positive/total (n)	Crude SOX10 positivity (%)	Pooled estimate of SOX10 positivity (95% CI)	Heterogeneity I^2^	*P*-value	Diagnostic implication
Pleomorphic adenoma (PA)	7	163/174	93.7	95.8% (91.7–99.9)	52.4%	0.050	Excellent marker
Basal cell adenoma (BCA)	4	20/21	95.2	91.3% (80.2–102.5)	0%	0.906	Very strong expression
Basal cell adenocarcinoma (BCAC)	2	13/15	86.6	84.8% (67.3–102.3)	0%	0.738	Strong but limited data
Adenoid cystic carcinoma (AdCC)	9	176/181	97.2	96.6% (94.1–99.2)	0%	0.982	Very high and consistent expression
Myoepithelioma	3	10/14	71.4	75.2% (52.0–98.4)	23.5%	0.271	High expression
Myoepithelial carcinoma	2	2/2	100	75.0% (15.0–100.0)	0%	0.999	Very small sample
Epithelial–myoepithelial carcinoma (EMC)	3	9/9	100	89.0% (71.6–106.5)	0%	0.872	Strong positivity (limited data)
Polymorphous adenocarcinoma (PAC)	3	32/33	97.0	97.4% (92.3–102.6)	0%	0.502	Excellent marker

Seven studies reported PA SOX10 immunohistochemistry, with 174 samples studied. Of these, 163 were SOX10 positive, corresponding to a crude observed positivity rate of 93.7%. The pooled meta-analytic estimate of SOX10 positivity was 95.8% (Suppl. Figure 1a). Correspondingly, SOX10 is an excellent marker of PA.

Four studies featured BCAs, and 21 tumors were studied. Of these, 20 were SOX10 positive, corresponding to a crude observed positivity rate of 95.2% and a pooled meta-analytic estimate of SOX10 positivity of 91.3% (Suppl. Figure 1b). SOX10 expression was very strong in the BCA.

Only two series studied BCACs. Thirteen of the 15 tumors studied were SOX10 positive, corresponding to a crude observed positivity rate of 86.7%. The pooled meta-analytic estimate of SOX10 positivity was 84.8% (Suppl. Figure 1c). SOX10 expression can be considered very strong, though it is based on limited data in BCAC.

AdCC was a focus in nine studies, with 181 immunohistochemistry results available. SOX10 was positive in 176 samples, corresponding to a crude observed positivity rate of 97.2%. The pooled meta-analytic estimate of SOX10 positivity was 96.6% (Suppl. Figure 1d). SOX10 expression in AdCC is high and consistent.

Three studies featured myoepithelioma, with 14 tumors studied. Of these, 10 were SOX10 positive, with a crude observed positivity rate of 71.4% and a pooled meta-analytic estimate of SOX10 positivity being 75.2% (Suppl. Figure 1e). Accordingly, SOX10 expression in myoepithelioma was high.

Myoepithelial carcinomas were covered in two studies, with two tumors studied overall. Both were SOX10 positive, corresponding to a crude observed positivity rate of 100%. However, the pooled meta-analytic estimate of SOX10 positivity was 75% (Suppl. Figure 1f). The pooled estimate reflects the meta-analytic model and should not be interpreted as the crude observed proportion. Although both tumors were SOX10 positive and heterogeneity was low, the findings were based on a very small sample size.

EMC was featured in three studies, and nine tumors were studied overall. All were SOX10 positive, with a crude observed positivity rate of 100% and a pooled meta-analytic estimate of SOX10 positivity of 89% (Suppl. Figure 1g). Similarly to myoepithelial carcinoma, the difference between the crude observed positivity rate and meta-analytic estimate of SOX10 positivity reflects the meta-analytic model. SOX10 positivity in EMC is strong but based on limited data.

PAC was studied in three articles comprising 33 tumors. In total, 32 were SOX10 positive, corresponding to a crude observed positivity rate of 97.0% and the pooled meta-analytic estimate of SOX10 positivity was 97.4% (Suppl. Figure 1h). Hence, SOX10 is an excellent marker of PAC.

### Salivary Gland Tumors with Oncocytic Features

Warthin’s tumor (WT), oncocytoma, acinic cell carcinoma (AciCC), secretory carcinoma (SC), mucoepidermoid carcinoma (MEC), and salivary duct carcinoma (SDC) are considered salivary gland tumors with oncocytic features. Table 2 presents the results of this group.

**Table 2:** Summary of SOX10 expression and diagnostic application in salivary gland tumors with oncocytic features

Tumor type	Studies (n)	SOX10 positive/total (n)	Crude SOX10 positivity (%)	Pooled estimate of SOX10 positivity (95% CI)	Heterogeneity I^2^	*P*-value	Diagnostic implication
Warthin tumor (WT)	3	0/36	0	2.3% (0–7.0)	0%	0.704	Typically negative → strong differential value
Oncocytoma	5	0/19	0	8.0% (− 2.7–18.7)	0%	0.944	Typically negative
Acinic cell carcinoma (AciCC)	7	92/95	96.8	97.4% (94.1–100.6)	7.7%	0.370	Reliable, strong expression
Secretory carcinoma (SC)	9	57/68	83.8	79.9% (55.1–104.7)	95.6%	<0.001	Considerable variability
Mucoepidermoid carcinoma (MEC)	9	28/175	16.0	20.2% (2.8–37.6)	95.1%	<0.001	Mostly negative
Salivary duct carcinoma (SDC)	8	0/63	0	3.7% (− 0.6–8.1)	0%	0.981	Typically negative

WT was featured in three studies, with a total of 36 tumors. None was SOX10 positive, corresponding to a crude observed positivity rate of 0% and making the pooled meta-analytic estimate of SOX10 positivity 2.3% (Suppl. Figure 2a). SOX10 can be considered typically negative in WT, giving it a high value in the differential diagnostics of salivary gland tumors with oncocytic features.

Oncocytomas were featured in only five studies, with a total of 19 tumors. None was SOX10 positive, corresponding to a crude observed positivity rate of 0%. The pooled meta-analytic estimate of SOX10 positivity in oncocytoma was 8% (Suppl. Figure 2b). The pooled estimate reflects the meta-analytic model and should not be interpreted as the crude observed proportion. Consequently, oncocytoma is typically negative in SOX10 immunohistochemistry.

AciCC was targeted in seven studies, with 95 tumors in total. Of these, 92 tumors were SOX10 positive, corresponding to a crude observed positivity rate of 96.8% and a pooled meta-analytic estimate of SOX10 positivity of 97.4% (Suppl. Figure 2c). Therefore, SOX10 expression in AciCC is reliable and strong.

SC was investigated in nine studies with 68 tumors in total. Of these, 57 tumors were SOX10 positive, corresponding to a crude observed positivity rate of 83.8%. The pooled meta-analytic estimate of SOX10 positivity was 79.9% (Suppl. Figure 2d). SC demonstrated considerable variability in SOX10 staining. No histological subtype or grade was stated, leaving the reason for the variability unclear.

MEC was featured in nine studies with 175 tumors in total. Of these, 28 tumors were SOX10 positive, corresponding to a crude observed positivity rate of 16.0%. The pooled meta-analytic estimate of SOX10 positivity was 20.2% (Suppl. Figure 2e). SOX10 was mostly negative in MEC.

SDC was the target of eight studies, with 63 tumors in total. None was SOX10 positive, corresponding to a crude observed positivity rate of 0%. The pooled meta-analytic estimate of SOX10 positivity was 3.7% (Suppl. Figure 2f). Typically, SDC can be considered SOX10 negative.

### Salivary Gland Tumors with Clear Cell Features

Myoepitheliomas, myoepithelial carcinomas, AciCCs, MECs, and hyalinizing clear cell carcinomas (HCCCs) are salivary gland tumors with clear cell features. Due to morphological heterogeneity, the following entities can be classified as salivary gland tumors with basaloid or oncocytic features: myoepitheliomas, myoepithelial carcinomas, AciCCs, and MECs. The results of these tumors were described earlier with salivary gland tumors having basaloid or oncocytic features. Table 3 summarizes the results of salivary gland tumors with clear cell features.

**Table 3:** Summary of SOX10 expression and diagnostic application in salivary gland tumors with clear cell features

Tumor type	Studies (n)	SOX10 positive/total (n)	Crude SOX10 positivity (%)	Pooled estimate of SOX10 positivity (95% CI)	Heterogeneity I^2^	*P*-value	Diagnostic implication
Myoepithelioma	3	10/14	71.4	75.2% (52.0–98.4)	23.5%	0.271	High expression
Myoepithelial carcinoma	2	2/2	100	75.0% (15.0–100.0)	0%	0.999	Very small sample
Acinic cell carcinoma (AciCC)	7	92/95	96.8	97.4% (94.1–100.6)	7.7%	0.370	Reliable, strong expression
Mucoepidermoid carcinoma (MEC)	9	28/175	16.0	20.2% (2.8–37.6)	95.1%	<0.001	Mostly negative
Hyalinizing clear cell carcinoma (HCCC)	3	2/24	8.3	8.8% (− 1.9–19.6)	0%	0.641	Mostly negative

HCCCs were featured in three studies with 24 tumors in total. Two were SOX10 positive, corresponding to a crude observed positivity rate of 8.3% and making the pooled meta-analytic estimate of SOX10 positivity 8.8% (Suppl. Figure 3). With these findings, HCCC can be considered mostly SOX10 negative.

### Other Analyzed Entities

Adenocarcinomas not otherwise specified (NOS) were researched in four studies, with nine tumors in total. Four were SOX10 positive, corresponding to a crude observed positivity rate of 44.4% and a pooled meta-analytic estimate of SOX10 positivity of 39.8% (Suppl. Figure 4a). Due to the variable results, the utility of SOX10 in the diagnostics of adenocarcinoma NOS is limited. Table 4 summarizes the other analyzed entities.

**Table 4:** Summary of SOX10 expression and diagnostic application in other analyzed entities

Tumor type	Studies (n)	SOX10 positive/total (n)	Crude SOX10 positivity (n, %)	Pooled estimate of SOX10 positivity (95% CI)	Heterogeneity I^2^	*P*-value	Diagnostic implication
Adenocarcinoma, NOS	4	4/9	44.4	39.8% (− 4.3–84.0)	78.7%	0.003	Very variable; limited utility
Canalicular adenoma (CA)	2	62/62	100	98.9% (96.4–101.4)	0%	0.575	Essentially universally positive
Intraductal carcinoma– combined	5	15/29	51.7	56.4% (17.6–95.1)	88.2%	<0.001	Strong heterogeneity → subtype-specific reporting recommended
Intraductal carcinoma– intercalated type	3	11/11	100	90.8% (75.9–105.8)	0%	0.854	Strong positivity
Intraductal carcinoma– apocrine type	2	0/6	0	10.3% (− 10.4–31.1)	0%	0.610	Typically negative
Microsecretory carcinoma	2	16/16	100	96.1% (87.2–105.0)	0%	0.545	Very high expression
Sialadenoma papilliferum (SP) (all subtypes)	2	10/20	50.0	50.0% (0–100)	0%	1.000	Insufficient evidence

Canalicular adenomas (CAs) were the focus of two studies with 62 tumors in total. All were SOX10 positive, corresponding to a crude observed positivity rate of 100%, and the pooled meta-analytic estimate of SOX10 positivity was 98.9% (Suppl. Figure 4b). As such, SOX10 is essentially universally positive in CA.

All types of intraductal carcinoma were featured in five studies, with 29 tumors in total. Fifteen were SOX10 positive, corresponding to a crude observed positivity rate of 51.7%. The pooled meta-analytic estimate of SOX10 positivity was 56.4% (Suppl. Figure 4c). Two subtypes of intraductal carcinoma had sufficient data for further analysis. The first was the intercalated duct type, which was examined in three studies with 11 tumors in total. All were SOX10 positive, corresponding to a crude observed positivity rate of 100% and a pooled meta-analytic estimate of SOX10 positivity of 90.8% (Suppl. Figure 4d). The other subtype analyzed was the apocrine type, which was evaluated by two studies with six tumors in total. None was SOX10 positive, corresponding to a crude observed positivity rate of 0%. The pooled meta-analytic estimate of SOX10 positivity was 10.3% (Suppl. Figure 4e). In conclusion, SOX10 is a strong marker of the intercalated subtype of intraductal carcinomas and is mostly negative in the apocrine subtype. These findings demonstrate the necessity of subtype-specific reporting in intraductal carcinomas.

Microsecretory carcinomas were featured in two studies, with 16 tumors in total. All were SOX10 positive, corresponding to a crude observed positivity rate of 100%, and the pooled meta-analytic estimate of SOX10 positivity was 96.1% (Suppl. Figure 4f). These findings imply that the expression of SOX10 in microsecretory carcinomas is very high.

Sialadenoma papilliferum (SP) was analyzed regarding all the subtypes combined. Two studies assessed them, with 20 tumors in total. Ten were SOX10 positive, corresponding to a crude observed positivity rate of 50%, and the pooled meta-analytic estimate of SOX10 positivity was 50% (Suppl. Figure 4g). The evidence was insufficient to draw any conclusions of SOX10 positivity regarding SP when analyzed with all subtypes combined.

### Entities not Analyzed

Meta-analyses of SOX10 positivity were not conducted on tumors that were not featured in at least two independent studies. Therefore, the evaluation of SOX10 positivity in these entities is based on very limited evidence, and their SOX10 positivity is summarized only in Suppl. Table 3.

### Cytological Samples

Only Schmitt et al. [10] evaluated cytological samples. The material used was a cell block. The findings of this study have been analyzed with histologic samples in forest plot construction. Eight of 13 samples of AciCC were SOX10 positive. They were stained in a nuclear pattern, and the percentage of cells stained was 50%. WT, MEC, and oncocytoma were SOX10 negative (0/16, 0/3, and 0/3, respectively). Polyclonal antibody was used for all cytological samples.

### SOX10 Staining Pattern, Cell Types, and Intensity

In all cases where the SOX10 staining pattern was explicitly stated, it was nuclear. A nuclear SOX10 staining pattern was reported in 29 studies (83%). No membranous or cytoplasmic staining was reported.

Abluminal cell positivity was reported in 82 samples in total, of which 61 were AdCC, 13 were canalicular adenoma-like PA, and eight were BCA [14, 31]. Luminal and abluminal cell positivity was found in 22 samples of AdCC, five samples of EMC, and three samples of AciCC [22, 23]. Luminal epithelial cell positivity was reported in 21 samples of sclerosing polycystic adenoma [30]. Canalicular cell positivity was reported in 16 samples of canalicular-like PA [33]. Endophytic ductal cell positivity was reported in 10 samples of SP [19, 27]. Chondroid, epitheloid, spindle, and plasmacytoid cell positivity was found in 10 samples of PA. However, the exact number of each cell type stained was not stated [22]. Epithelial and mesenchymal cell positivity was reported in nine samples of PA, and in the same study, epithelial and myoepithelial positivity was reported in three samples of EMC [6]. Acinar cell positivity was found in nine samples of the mucoacinar subtype of MEC [40]. Polygonal cell positivity was found in six samples of MEC [6]. Spindle cell positivity was reported in four samples of myoepithelioma and one sample of myoepithelial carcinoma [22]. Intercalated duct cell positivity was found in two, and a combination of intercalated duct and oncocytic cell positivity was noticed in three samples of intraductal carcinoma [16]. SOX10 positivity in serous acini and mucous acini was reported in two HCCC samples [26]. In most studies of the meta-analysis, the text did not explicitly state the exact cell type stained positive for SOX10.

Information on SOX10 staining intensity and the SOX10 staining percentage in the analyzed publications was equally rare. High staining intensity was reported in PA [22], SP [19, 27], hybrid and intercalated duct types of intraductal carcinoma [16], EMC [22], SC [11, 36], AdCC [9], PAC [13], MEC [29], striated duct adenoma [18], AciCC [22], CA [8], microsecretory adenocarcinoma [15], myoepithelial carcinoma, and myoepithelioma [22]. A variable, high to low intensity, was reported in AdCC [12, 13, 22]. Intermediate staining intensity was reported in hybrid intraductal carcinoma [16], while low staining intensity was reported in HCCC [26]. SOX10 staining percentages were rarely reported as exact percentages, and the range of percentages in SOX10 positive cases ranged from 10%–100%.

#### SOX10 Antibodies Used

In terms of antibody source and dilution, nine studies used Cell Marque polyclonal antibody (Rocklin, CA, USA) [10, 12, 23, 29, 36] or the following clones, if stated: EP268 [11], SP267 [30], and Catalog #PA0813 [13]. Four studies used the Ventana Medical Systems (Tucson, AZ, USA) antibody, which was ready to use. The clone used was SP267 [31] or was not stated [15, 17, 20]. Four studies used Bio SB (Santa Barbara, CA, USA) antibody, which was ready to use, and all used clone EP268 [6, 27, 28, 39]. Four studies used Santa Cruz Biotechnology’s (Santa Cruz, CA, USA) antibody with 1:200 and 1:500 dilutions, and the clones used were N-20 [8, 22, 26] and A-2 (sc-365692) [25]. Two studies used Abcam (Cambridge, UK) antibody with the dilutions 1:100 [34] and 1:25 [38], with the former using the clone SP267. Two studies used DCS polyclonal antibodies with a 1:25 dilution [32, 33]. Two studies used the Biocare Medica predilute antibody (clone BC34) [19, 40]. One study used Leica Biosystems (CA, USA) with the clone BC34 and an unknown dilution [14]. One study used Diagnostic BioSystems’ (Pleasanton, CA, USA) antibody with 1:100 dilution and the clone EP268 [24]. One study used a Biocare antibody with a 1:100 dilution and the clone BC34 [16]. The source, clone, and dilution were not stated in five (14%) studies [18, 21, 35, 37, 41]. The different antibodies used are tabulated in Suppl. Table 4.

## Discussion

In this meta-analysis of 35 studies, 32 histological entities and 1146 histological samples in total, along with 35 cytological samples, were evaluated for SOX10 immunoexpression. SOX10 showed a pooled meta-analytic positivity estimate of 95.8% in PA, making it an excellent marker for PA. PA was explicitly stated as SOX10 positive by both the WHO Classification of Tumors and *Diagnostic Immunohistochemistry* [1, 5]. In this meta-analysis, five studies reported 100% SOX10 positivity in PA [6, 21, 22, 31, 39]. In contrast, the lowest PA SOX10 positivity (76%) was found in Ko et al. [14]. Only this study evaluated SOX10 positivity in PA with the clone BC34. These findings entrench PA as a SOX10-positive tumor.

PAC is another entity in which SOX10 is an excellent marker (pooled estimate of SOX10 positivity 97.4%, 95% CI 92.3–102.6%). 95% confidence interval exceeding 100% reflects the meta-analytic model used and should not be interpreted as a biologically possible result. Unlike PA, PAC was stated as SOX10 positive by *Diagnostic Immunohistochemistry* [5], while WHO Classification of Tumors did not address the SOX10 positivity of PAC. Of the three studies in this meta-analysis, two reported total SOX10 positivity [13, 39]. Conversely, one study had only a single sample of SOX10-negative PAC [14]. As was the case with PA, the SOX10 clone used in the study was BC34. The other studies used EP268 [39] and Cell Marque (Rocklin, CA, USA) Catalog #PA0813 [13].

CA can be considered essentially universally SOX10 positive, with a crude observed positivity rate of 100%. The pooled meta-analytic estimate of SOX10 positivity in CA was 98.9% (95% CI 96.4–101.4%). As a result, there was no disparity in SOX10 positivity between the SOX10 clones.

Several entities had strong and consistent SOX10 immunoexpression. BCA (pooled estimate 91.3%, 95% CI 80.2–102.5%) had total SOX10 positivity in two studies using the EP268 clone [6, 39] and in one study using an unknown clone [21]. In contrast, eight out of nine samples of BCA were SOX10 positive with the BC34 clone [14]. The result should be interpreted with caution given that the sample size was small. AdCC also had strong SOX10 expression, with a pooled estimate of SOX10 positivity of 96.6% (95% CI 94.1–99.2%). Unlike PA, PAC, and BCA, there was no distinction between BC34 and the other clones used. Indeed, the polyclonal antibody used by Yang et al. [12] exhibited the lowest SOX10 positivity among studies featuring AdCC (88%). All cases of myoepithelioma (pooled meta-analytic estimate 75.2%, 95% CI 52.0–98.4%) were SOX10 positive when evaluated with the clone N-20 [22] and an unknown clone [21]. However, only 56% were SOX10 positive for the BC34 clone [14]. AciCC (pooled estimate of SOX10 positivity 97.4%, 95% CI 94.1–100.6%) had a crude observed SOX10 positivity rate of 96.8% and had total positivity across six studies [6, 10, 14, 22, 38, 39]. Nonetheless, only 50% were SOX10 positive in one study using a polyclonal SOX10 antibody [23]. However, Schmitt et al. [10] used the same antibody with 26 samples. With no information on the tumor grade or subtype, the reason for this difference is unknown. All samples of the intercalated subtype of intraductal carcinoma (crude observed positivity rate 100%, meta-analytic estimate 90.8%, 95% CI 75.9–105.8%) and microsecretory carcinoma (crude observed positivity rate 100%, meta-analytic estimate 96.1%, 95% CI 87.2–105.0%) were SOX10 positive. Confidence intervals exceeding 100% reflect the meta-analytic model and should not be interpreted as biologically possible results.

Strong positivity based on limited data was found in BCAC (pooled estimate 84.8%, 95% CI 67.3–102.3%) and EMC (pooled estimate 89.0%, 95% CI 71.6–106.5%), with no variations between the clones used. The crude observed SOX10 positivity rate of myoepithelial carcinoma was 100%. This finding was based on a very small sample size of two cases. There was no difference between the clones used [22, 39].

WT is essentially always SOX10 negative, with a crude observed positivity rate of 0% (pooled meta-analytic estimate 2.3%, 95% CI 0–7.0%), giving the marker a strong differential diagnostic value. The apocrine type of intraductal carcinoma (pooled estimate 10.3%, 95% CI − 10.4–31.1%), SDC (pooled estimate 3.7%, 95% CI − 0.6–8.1%), and oncocytoma (pooled estimate 8.0%, 95% CI − 2.7–18.7%) were also SOX10 negative, with a crude observed SOX10 positivity rate of 0% in all of them. In SDC, the only positive samples were low grade [6], likely representing a historical entity and thus not included in the meta-analysis. MEC can be considered mostly negative (pooled estimate 20.2%, 95% CI 2.8–37.6%), although with a crude observed positivity of 16.0% and a pooled estimate of 20.2% almost one-fifth of them are SOX10 positive. As 100% of mucoacinar subtypes and 30% of oncocytic subtypes of MEC were SOX10 positive, the subtype explains results’ variability.

Entities with considerable variability in SOX10 expression include SC (pooled estimate 79.9%, 95% CI 55.1–104.7%) and adenocarcinoma not otherwise specified (pooled estimate 39.8%, 95% CI − 4.3–84%). In SC, no subtypes or grades were reported, and the results within the SOX10 clones varied. In adenocarcinoma not otherwise specified, the only positive samples were the four studied by Dababneh et al. [21]. The SOX10 clone was not reported, and one of the entities was described as intermediate grade and the other as low grade. There was also strong heterogeneity in the SOX10 expression of intraductal carcinomas when both subtypes were combined (pooled estimate 56.4%, 95% CI 17.6–95.1%). As noted by Hsieh et al. [28], SOX10 is a strong marker of the intercalated subtype of intraductal carcinoma and is mostly negative in the apocrine subtype. This meta-analysis provides further proof of these findings and demonstrates the necessity of subtype-specific reporting in intraductal carcinomas. SOX10 expression in SP (pooled estimate 50.0%, 95% CI 0–100%) remains unclear due to insufficient data. SP was evaluated with EP268 [27] and BC34 [19], and both studies had variable results. In Hsieh et al., the oncocytic (n = 4) and exophytic (n = 5) subtypes of SP were SOX10 negative. Nine other entities were SOX10 positive. Similarly, the oncocytic SP immunostained by Owosho et al. was SOX10 negative; the other SP sample was SOX10 positive.

The differences in staining results between SOX10 antibody clones are generally negligible. However, as discussed earlier, some were found. EP268, SP267, N-20, A-2, and polyclonal antibodies produce similar results. In contrast, BC34 provides smaller percentages of SOX10-positive tumors, especially in basaloid salivary gland tumors. Regrettably, information on SOX10 staining intensity and the percentage of SOX10-positive cells was scarce. Thus, no conclusions could be drawn about the effect of the SOX10 clone on these parameters.

In the small series of cytological SOX10 stains, WT, MEC, and oncocytoma were SOX10 negative, adhering to the findings in the histological samples. However, only 62% of AciCC were SOX10 positive, highlighting a stark difference compared to the 97.4% pooled proportion of histological samples. This finding mirrors the common lowered immunopositivity in cytological preparations caused by fixation and processing differences [42–44]. The antibody used in the cytological samples was polyclonal, and the staining results are in line with the histological counterparts stained with polyclonal antibody. Of note, in histological samples, the only antibody with negative SOX10 results in AciCC was polyclonal.

To conclude the results of the meta-analysis, the list of entities likely to be SOX10 positive includes PA, BCA, BCAC, AdCC, myoepithelioma, myoepithelial carcinoma, EMC, PAC, AciCC, CA, intercalated type of intraductral carcinoma and microsecretory carcinoma. The list of entities likely to be SOX10 negative includes WT, oncocytoma, MEC, SDC, HCCC and apocrine type of intraductal carcinoma.

Although morphologic groups have overlap between each other, certain observations can be made. SOX10 tends to be positive in all entities which are likely to have basaloid morphology, limiting the usefulness of SOX10 in differentiating them from each other. In salivary gland tumors which primarily present as oncocytic, SOX10 can only be considered positive in AciCC with the rest having either high variability in results or being SOX10 negative. Salivary gland tumors with clear cell features form a heterogenous group with considerable overlap between the other groups. HCCC is mostly SOX10 negative, which helps differentiate it from myoepithelioma, myoepithelial carcinoma and AciCC. In contrast, MEC is SOX10 negative as well, limiting the usefulness of SOX10 in differentiating between HCCC and MEC.

Of the entities not further analyzed, all samples of sialoblastoma, carcinoma-ex PA, poorly differentiated carcinoma, and sclerosing polycystic adenoma were SOX10 positive. Most cases of striated duct adenoma were SOX10 positive. Only a minor portion of carcinoma NOS, SDC ex-PA, and adenocarcinoma ex-PA NOS was SOX10 positive. All samples of cribriform cystadenocarcinoma, papillary cystadenoma, lymphoepithelial carcinoma, squamous cell carcinoma, and carcinosarcoma were SOX10 negative. Due to very limited evidence, these results should be approached with caution. More research should be conducted to confirm these findings.

The most significant strength of this study lies in its large amount of data, with an analysis of 35 studies and 1183 tumors immunostained with SOX10. Twenty of the 32 histological entities were eligible for meta-analysis and forest plot construction. The weaknesses of this study stem from the use of only one database, PubMed, limiting the number of studies available for screening. Additionally, it is possible for a study to not contain the search term “SOX10” in the title, abstract or keywords, thus making it invisible in the literature search. Dozens of case reports and five studies with inconsistencies were excluded, which also limits the available data for the meta-analysis. These limitations were apparent in the original study’s design, where the number of articles proved to be insufficient for meta-analysis. Furthermore, the original study’s design would have suffered from confirmation bias due to only including the search words of entities with known SOX10 positivity. This weakness was eliminated by this study’s design, which used one search term. As non-English articles were excluded, several promising articles written in the Chinese language were excluded.

In conclusion, SOX10 is a strong and reliable marker of basaloid salivary gland tumors. However, some entities in the groups of tumors with oncocytic features or clear cell features are SOX10 immunopositive. This meta-analysis supplies a comprehensive list of SOX10-positive and -negative entities, while illuminating the need for more research on entities for which SOX10 positivity could not be discerned due to insufficient data. Furthermore, the findings highlight the necessity of subtype-specific reporting and the reporting of the antibody clone in immunohistochemical studies.

## Supplementary Information

Below is the link to the electronic supplementary material.

Supplementary Material 1

## Data Availability

The dataset used to support the findings of this study is available from the corresponding author at ivana.kholova@tuni.fi upon reasonable request.

## Code Availability

No custom code was developed or used in this study. Statistical analyses were performed using OpenMeta [Analyst] Version 0.24.1, while Microsoft Excel was used for data tabulation.
