# Supplementary material for: SOX10 Expression in Primary Salivary Gland Tumors: A Meta-Analysis
Source: Head Neck Pathol. 2026 Oct 16;20(1):104. doi: 10.1007/s12105-026-01975-3 (PMC13675452; doi:10.1007/s12105-026-01975-3)
Supplement: Supplementary file 1 [file 12105_2026_1975_MOESM1_ESM.docx]

**SOX10 Expression in Primary Salivary Gland Tumors: A Meta-Analysis**

**Otto Tahvanainen^1,2^, Henri Lagerstam^3,4^, David Kalfert^5^, Ivana Kholová^1,3,4^**

**^1^Institute of Clinical Medicine, University of Eastern Finland, Kuopio, Finland.**

**^2^Kuopio University Hospital, Kuopio, Finland.**

**^3^Faculty of Medicine and Health Technology, Tampere University, Tampere, Finland.**

**^4^Fimlab Laboratories, Pathology, Tampere, Finland.**

**^5^Department of Otorhinolaryngology, Head and Neck Surgery, First Faculty of Medicine, University Hospital Motol and Homolka, Charles University, Prague, Czech Republic.**

**Corresponding author: Ivana Kholová, ivana.kholova@tuni.fi**

**Supplementary Data**

Supplementary Table 1: Search keywords used for the first PubMed literature search. The second search did not include cytology or FNA key words.

| **“SOX10 and ___ and (cytology or FNA)”** | |
| --- | --- |
| Salivary gland | Sialadenoma papilliferum |
| Parotid gland | Sclerosing polycystic adenoma |
| Submandibular gland | Acinic cell carcinoma |
| Sublingual gland | Secretory carcinoma |
| Pleomorphic adenoma | Microsecretory adenocarcinoma |
| Myoepithelioma | Intraductal carcinoma |
| Canalicular adenoma | Myoepithelial carcinoma |
| Cystadenoma | Sialoblastoma |
| Basal cell adenoma | Polymorphous adenocarcinoma |
| Adenoid cystic carcinoma | Epithelial-myoepithelial carcinoma |

Supplementary Table 2: An overview of the studies chosen for meta-analysis.

| PMID | 1^st^ author | Year of Publication | Country of Origin | Continent | Institutional/Multi-institutional/International | Period of Study (years) | University hospital/Regional hospital/Private lab/Publicly owned private laboratory | Total number of samples studied |
| --- | --- | --- | --- | --- | --- | --- | --- | --- |
| 23558573 | Ohtomo R | 2013 | Japan | Asia | Institutional | 1998 to 2010 (12 years) | Regional hospital | 90 |
| 25141970 | Thompson LDR | 2015 | USA | North America | Multi-institutional | 1986 to 2012 (26 years) | Regional hospital, University hospital | 67 |
| 25456394 | Urano M | 2015 | Japan | Asia | Institutional | 1993 to 2012 (19 years) | University hospital | 18 |
| 26076064 | North JP | 2015 | USA | North America | Institutional | ND | University hospital | 12 |
| 26619208 | Schmitt AC | 2015 | USA | North America | Institutional | ND | University hospital | 110 |
| 27327192 | Hsieh MS | 2016 | Republic of China | Asia | Institutional | 1999-2015 (16 years) | University hospital | 142 |
| 27658560 | Ni H | 2016 | People's republic of China | Asia | Institutional | 1997-2014 (17 years) | University hospital | 89 |
| 28548128 | Krings G | 2017 | USA | North America | Multi-institutional | 2006-2015 (9 years) | University hospital, Regional hospital | 6 |
| 28781197 | Baghai F | 2017 | Iran | Asia | Multi-institutional | 2004-2014 (10 years) | Regional hospital | 111 |
| 29434341 | Takamatsu M | 2018 | Japan | Asia | Institutional | ND | Regional hospital | 3 |
| 30424592 | Lee JH | 2018 | Republic of Korea | Asia | Institutional | 2007 - March 2017 (10 years) | Regional hospital | 113 |
| 31105427 | Yang C | 2019 | USA | North America | Multi-institutional | 1992-2014 (22 years) | University hospital, Regional hospital | 17 |
| 31222589 | Adkins BD | 2020 | USA | North America | Multi-institutional | 1994-2015 (21 years) | University hospital | 50 |
| 31473937 | Hsieh MS | 2020 | Republic of China | Asia | International | 1994-2018 (24 years) | University hospital, Regional hospital | 18 |
| 31895100 | Ko YCK | 2020 | USA | North America | Institutional | ND | University hospital | 173 |
| 32383006 | Hsieh MS | 2020 | Republic of China | Asia | Institutional | 1999-2018 (19 years) | University hospital | 33 |
| 33002921 | Skálová A | 2020 | Czech Republic | Europe | Institutional | 1996-2019 (23 years) | Private lab | 125 |
| 33763743 | Luna-Ortiz K | 2021 | Mexico | North America | Institutional | 1996-2018 (22 years) | Regional hospital | 11 |
| 33871952 | Agaimy A | 2021 | Germany | Europe | Multi-institutional | ND | University hospital, Regional hospital | 4 |
| 33982215 | Bishop JA | 2021 | USA | North America | International | ND | University hospital, Regional hospital | 24 |
| 33991527 | Fisch AS | 2021 | USA | North America | Multi-institutional | 2014-2021 (7 years) | Regional hospital | 11 |
| 34091485 | Bundele M | 2021 | Singapore | Asia | International | ND | Regional hospital, University hospital | 11 |
| 34324456 | Agaimy A | 2022 | Germany | Europe | International | ND | Regional hospital, University hospital, Private lab | 28 |
| 34510113 | Skálová A | 2022 | Czech Republic | Europe | Multi-institutional | ND | University hospital, Private lab | 36 |
| 34985683 | McLean-Holden AC | 2022 | USA | North America | Multi-institutional | ND | University hospital, Regional hospital | 10 |
| 36221318 | Weinreb I | 2023 | Canada | North America | ND | ND | ND | 4 |
| 36510691 | Rooper LM | 2023 | USA | North America | International | ND | University hospital, Regional hospital | 12 |
| 36632022 | Owosho AA | 2023 | USA | North America | Institutional | 2001-2021 (20 years) | University hospital | 8 |
| 38935197 | Delfin L | 2024 | USA | North America | International | ND | University hospital, Regional hospital | 7 |
| 39101978 | Mahomed F | 2024 | South Africa | Africa | Institutional | 2017-2023 (6 years) | University hospital | 7 |
| 39306518 | de Aquino SN | 2025 | Brazil | South America | Institutional | 2014-2024 (10 years) | University hospital | 10 |
| 39360582 | Dababneh MN | 2024 | USA | North America | Multi-institutional | 2013-2023 (10 years) | Regional hospital, University hospital | 21 |
| 39560855 | Kobayashi K | 2024 | Japan | Asia | Institutional | 2007-2020 (13 years) | Regional hospital | 41 |
| 39617100 | Sakyo A | 2025 | Japan | Asia | Institutional | 2018-2023 (5 years) | Regional hospital | 12 |
| 40033554 | Klubíčková N | 2025 | Czech Republic | Europe | Institutional | ND (22 years) | Private lab | 62 |

Supplementary table 3: A summary of entities not analyzed.

| **Tumor type** | **Study** | **SOX10 positivity (positive/total, %)** |
| --- | --- | --- |
| Carcinoma not otherwise specified | Ohtomo R et al. [22] | 5/22 (23%) |
| Sclerosing polycystic adenoma | Skálová A et al. [30] | 21/21 (100%) |
| Salivary duct carcinoma ex-pleomorphic adenoma | Kobayashi K et al. [25] | 1/6 (17%) |
| Lymphoepithelial carcinoma | Hsieh MS et al. [6] | 0/6 (0%) |
| Striated duct adenoma | Rooper LM et al. [18] | 4/5 (80%) |
| Adenocarcinoma ex-pleomorphic adenoma not otherwise specified | Kobayashi K et al. [25] | 1/4 (25%) |
| Cribriform cystadenocarcinoma | Urano M et al. [23] | 0/2 (0%) |
| Sialoblastoma | Hsieh MS et al. [6] | 1/1 (100%) |
| Carcinoma ex-pleomorphic adenoma | Lee JH et al. [39] | 1/1 (100%) |
| Papillary cystadenoma | Schmitt AC et al. [10] | 0/1 (0%) |
| Poorly differentiated carcinoma | Lee JH et al. [39] | 1/1 (100%) |
| Carcinosarcoma | Lee JH et al. [39] | 0/1 (0%) |

Supplementary table 4: Summary of SOX10 clones and positivity rates in selected salivary gland tumor entities. Depicted here are all entities featured in at least two independent studies which and data of at least one SOX10 antibody used. Due to the small sample sizes, differences between antibodies can most often be attributed to coincidence. BC34 tends to give negative results for PA more often than other antibodies, and similar results with smaller sample sizes can be seen in myoepithelioma.

|  | A-2 - Santa Cruz Biotechnology (Santa Cruz, CA, USA)  (SOX10 positive/total (%)) | BC34 – Biocare Medica; Leica Biosystems (CA, USA); Biocare  (SOX10 positive/total (%)) | Catalog #PA0813 – Cell Marque (Rocklin, CA, USA)  (SOX10 positive/total (%)) | EP268 - Cell Marque (Rocklin, CA, USA); Bio SB (Santa Barbara, CA, USA); Diagnostic Biosystems (Pleasanton, CA, USA)  (SOX10 positive/total (%)) | N-20 - Santa Cruz Biotechnology (Santa Cruz, CA, USA)  (SOX10 positive/total (%)) | Polyclonal antibody - Cell Marque (Rocklin, CA, USA); DCS  (SOX10 positive/total (%)) | SP267 - Cell Marque (Rocklin, CA, USA); Ventana Medical Systems (Tucson, AZ, USA); Abcam (Cambridge, UK)  (SOX10 positive/total (%)) |
| --- | --- | --- | --- | --- | --- | --- | --- |
| Pleomorphic adenoma | ND | 33/43 (76.7) | ND | 57/57 (100) | 10/10 (100) | 16/17 (94.1) | 40/40 (100) |
| Basal cell adenoma | ND | 8/9 (88.9) | ND | 11/11 (100) | ND | ND | ND |
| Basal cell adenocarcinoma | ND | ND | ND | 1/1 (100) | ND | ND | ND |
| Adenoid cystic carcinoma | 7/7 (100) | 61/63 (96.8) | 25/25 (100) | 16/16 (100) | 22/23 (95.7) | 15/17 (88.2) | ND |
| Myoepithelioma | ND | 5/9 (55.6) | ND | ND | 4/4 (100) | ND | ND |
| Myoepithelial carcinoma | ND | ND | ND | 1/1 (100) | 1/1 (100) | ND | ND |
| Epithelial-myoepithelial carcinoma | ND | ND | ND | 4/4 (100) | 5/5 (100) | ND | ND |
| Polymorphous adenocarcinoma | ND | 6/7 (85.7) | 25/25 (100) | 1/1 (100) | ND | ND | ND |
| Warthin’s tumor | ND | ND | ND | 0/27 (0) | 0/2 (0) | 0/7 (0) | ND |
| Oncocytoma | ND | 0/9 (0) | ND | 0/5 (0) | 0/2 (0) | 0/3 (0) | ND |
| Acinic cell carcinoma | ND | 7/7 (100) | ND | 30/30 (100) | 8/8 (100) | 29/32 (90.6) | ND |
| Secretory carcinoma | 2/2 (100) | 3/3 (100) | ND | 18/19 (94.7) | ND | 12/22 (54.5) | ND |
| Mucoepidermoid carcinoma | 0/9 (0) | 12/31 (38.7) | ND | 6/51 (11.8) | 0/6 (0) | 10/56 (17.9) | ND |
| Salivary duct carcinoma | 0/7 (0) | 0/3 (0) | ND | 2/35 (5.7) | 0/6 (0) | 0/3 (0) | ND |
| Hyalinizing clear cell carcinoma | ND | ND | ND | 0/12 (0) | 2/12 (16.7) | ND | ND |
| Adenocarcinoma NOS | 0/2 (0) | 0/1 (0) | ND | 0/2 (0) | ND | ND | ND |
| Canalicular adenoma | ND | ND | ND | ND | 52/52 (100) | ND | 10/10 (100) |
| Intraductal carcinoma – all subtypes | ND | 6/7 (85.7) | ND | 4/9 (44.4) | ND | 1/1 (100) | ND |
| Intraductal carcinoma – intercalated duct type | ND | 4/4 (100) | ND | 4/4 (100) | ND | 1/1 (100) | ND |
| Intraductal carcinoma – apocrine type | ND | 0/1 (0) | ND | 0/5 (0) | ND | ND | ND |
| Sialadenoma papilliferum | ND | 1/2 (50) | ND | 9/18 (50) | ND | ND | ND |

Footnotes:

ND=Not determined

NOS=Not otherwise specified

**Supplementary Fig. 1a** The pooled proportion of SOX-10 positive cases in pleomorphic adenoma (PA) was evaluated across 7 studies, comprising a total of 174 tumors with immunohistochemistry results available. SOX-10 positivity was detected in 163 out of 174 cases. The estimated pooled proportion of SOX-10 expression was 95.8% (95% CI: 91.7% to 99.9%). Statistical heterogeneity was moderate: I² = 52.44% (P = 0.050), indicating some variability among studies

**Supplementary Fig. 1b** The pooled proportion of SOX-10 positive cases in basal cell adenoma (BCA) was evaluated across 4 studies, comprising a total of 21 tumors with immunohistochemical assessment available. SOX-10 positivity was detected in 20 out of 21 cases. The estimated pooled proportion of SOX-10 expression was 91.3% (95% CI: 80.2% to 102.5%). Statistical heterogeneity was negligible: I² = 0% (P = 0.906), indicating consistent results across studies

**Supplementary Fig. 1c** The pooled proportion of SOX-10 positive cases in basal cell adenocarcinoma (BCAC) was assessed across 2 studies, comprising a total of 15 tumors with immunohistochemistry data available. SOX-10 positivity was confirmed in 13 out of 15 cases. The estimated pooled proportion of SOX-10 expression was 84.8% (95% CI: 67.3% to 102.3%). Statistical heterogeneity was negligible: I² = 0% (P = 0.738), indicating consistent results between studies

**Supplementary Fig. 1d** The pooled proportion of SOX-10 positive cases in adenoid cystic carcinoma (AdCC) was evaluated across 9 studies, comprising a total of 181 tumors with immunohistochemistry results available. SOX-10 positivity was observed in 176 out of 181 cases. The estimated pooled proportion of SOX-10 expression was 96.6% (95% CI: 94.1% to 99.2%). Statistical heterogeneity was negligible: I² = 0% (P = 0.982), indicating highly consistent findings across studies

**Supplementary Fig. 1e** The pooled proportion of SOX-10 positive cases in myoepithelioma was evaluated across 3 studies, comprising a total of 14 tumors with immunohistochemistry data available. SOX-10 positivity was observed in 10 out of 14 cases. The estimated pooled proportion of SOX-10 expression was 75.2% (95% CI: 52.0% to 98.4%). Statistical heterogeneity was low: I² = 23.46% (P = 0.271), indicating relatively consistent results across studies

**Supplementary Fig. 1f** The pooled proportion of SOX-10 positive cases in myoepithelial carcinoma was assessed across 2 studies, comprising a total of 2 tumors with immunohistochemistry results available. SOX-10 positivity was reported in both cases (2/2). The estimated pooled proportion of SOX-10 expression was 75.0% (95% CI: 15.0% to 100.0%). Statistical heterogeneity was absent: I² = 0% (P = 0.999), indicating consistent findings across studies, though based on very limited evidence. Pooled proportions represent random-effects meta-analytic estimates and may differ from crude proportions calculated from aggregated case numbers

**Supplementary Fig. 1g** The pooled proportion of SOX-10 positive cases in epithelial-myoepithelial carcinoma (EMC) was evaluated across 3 studies, comprising a total of 9 tumors with immunohistochemistry data available. SOX-10 positivity was observed in all 9 cases. The estimated pooled proportion of SOX-10 expression was 89.0% (95% CI: 71.6% to 106.5%). Statistical heterogeneity was absent: I² = 0% (P = 0.872), indicating highly consistent findings across studies. Pooled proportions represent random-effects meta-analytic estimates and may differ from crude proportions calculated from aggregated case numbers

**Supplementary Fig. 1h** The pooled proportion of SOX-10 positive cases in polymorphous adenocarcinoma (PAC) was evaluated across 3 studies, comprising a total of 33 tumors with immunohistochemistry results available. SOX-10 positivity was observed in 32 out of 33 cases. The estimated pooled proportion of SOX-10 expression was 97.4% (95% CI: 92.3% to 102.6%). Statistical heterogeneity was absent: I² = 0% (P = 0.502), indicating highly consistent findings across studies

**Supplementary Fig. 2a** The pooled proportion of SOX-10 positive cases in Warthin tumor (WT) was evaluated across 3 studies, comprising a total of 36 tumors with immunohistochemistry results available. SOX-10 positivity was detected in 0 out of 36 cases. The estimated pooled proportion of SOX-10 expression was 2.3% (95% CI: 0% to 7.0%). No heterogeneity was observed: I² = 0% (P = 0.704), indicating high consistency across studies. These findings confirm that SOX-10 expression is exceptionally rare in Warthin tumors. Pooled proportions represent random-effects meta-analytic estimates and may differ from crude proportions calculated from aggregated case numbers

**Supplementary Fig. 2b** The pooled proportion of SOX-10 positive cases in oncocytoma was evaluated across 5 studies, comprising a total of 19 tumors with immunohistochemistry results available. No SOX-10 positive tumors were identified (0/19). The estimated pooled proportion of SOX-10 expression was 8.0% (95% CI: –2.7% to 18.7%). Statistical heterogeneity was absent: I² = 0% (P = 0.944), indicating highly consistent results across studies. Pooled proportions represent random-effects meta-analytic estimates and may differ from crude proportions calculated from aggregated case numbers

**Supplementary Fig. 2c** The pooled proportion of SOX-10 positive cases in acinic cell carcinoma (AciCC) was evaluated across 7 studies, comprising a total of 95 AciCC tumors with immunohistochemical data available. SOX-10 positivity was confirmed in 92 out of 95 tumors. The estimated pooled proportion of SOX-10 expression was 97.4% (95% CI: 94.1% to 100.6%). Statistical heterogeneity was low: I² = 7.66% (P = 0.370)

**Supplementary Fig. 2d** The pooled proportion of SOX-10 positive cases in secretory carcinoma (SC) was evaluated across 9 studies, comprising a total of 68 tumors with immunohistochemistry results available. SOX-10 positivity was detected in 57 out of 68 cases. The estimated pooled proportion of SOX-10 expression was 79.9% (95% CI: 55.1% to 104.7%). Substantial heterogeneity was present: I² = 95.59% (P < 0.001), indicating considerable variability among studies

**Supplementary Fig. 2e** The pooled proportion of SOX-10 positive cases in mucoepidermoid carcinoma (MEC) was evaluated across 9 studies, comprising a total of 175 tumors with immunohistochemistry results available. SOX-10 positivity was observed in 28 out of 175 cases. The estimated pooled proportion of SOX-10 expression was 20.2% (95% CI: 2.8% to 37.6%). Statistical heterogeneity was substantial: I² = 95.06% (P < 0.001), indicating major variability among study findings

**Supplementary Fig. 2f** The pooled proportion of SOX-10 positive cases in salivary duct carcinoma (SDC) was evaluated across 8 studies, comprising a total of 63 tumors with immunohistochemistry results available. No SOX-10 positive tumors were identified (0/63). The estimated pooled proportion of SOX-10 expression was 3.7% (95% CI: –0.6% to 8.1%). Statistical heterogeneity was absent: I² = 0% (P = 0.981), indicating highly consistent findings across studies. Pooled proportions represent random-effects meta-analytic estimates and may differ from crude proportions calculated from aggregated case numbers. The two cases designated as low-grade salivary duct carcinoma by Hsieh et al. were not included in the conventional SDC analysis because this historical category may correspond to a distinct entity currently regarded within the intraductal carcinoma spectrum.

**Supplementary Fig. 3** The pooled proportion of SOX-10 positive cases in hyalinizing clear cell carcinoma (HCCC) was evaluated across 3 studies, comprising a total of 24 tumors with immunohistochemistry data available. SOX-10 positivity was observed in 2 out of 24 cases. The estimated pooled proportion of SOX-10 expression was 8.8% (95% CI: –1.9% to 19.6%). Statistical heterogeneity was absent: I² = 0% (P = 0.641), indicating consistent findings among studies

**Supplementary Fig. 4a** The pooled proportion of SOX-10 positive cases in adenocarcinoma not otherwise specified (Adenocarcinoma NOS) was evaluated across 4 studies, comprising a total of 9 tumors with immunohistochemical results available. SOX-10 positivity was observed in 4 out of 9 cases. The estimated pooled proportion of SOX-10 expression was 39.8% (95% CI: –4.3% to 84.0%). Statistical heterogeneity was high: I² = 78.74% (P = 0.003), indicating substantial variability among the included studies

**Supplementary Fig. 4b** The pooled proportion of SOX-10 positive cases in canalicular adenoma (CA) was assessed across 2 studies, comprising a total of 62 tumors with immunohistochemistry data available. SOX-10 positivity was detected in all 62 cases. The estimated pooled proportion of SOX-10 expression was 98.9% (95% CI: 96.4% to 101.4%). Statistical heterogeneity was absent: I² = 0% (P = 0.575), indicating highly consistent results across studies. Pooled proportions represent random-effects meta-analytic estimates and may differ from crude proportions calculated from aggregated case numbers

**Supplementary Fig. 4c** The pooled proportion of SOX-10 positive cases in intraductal carcinoma (all subtypes combined) was evaluated across 5 studies, comprising a total of 29 tumors with immunohistochemistry data available. SOX-10 positivity was reported in 15 out of 29 cases. The estimated pooled proportion of SOX-10 expression was 56.4% (95% CI: 17.6% to 95.1%). Statistical heterogeneity was high: I² = 88.19% (P < 0.001), indicating considerable variability among studies and subtypes

**Supplementary Fig. 4d** The pooled proportion of SOX-10 positive cases in intraductal carcinoma – intercalated duct type was evaluated across 3 studies, comprising a total of 11 tumors with immunohistochemistry results available. SOX-10 positivity was detected in all 11 cases. The estimated pooled proportion of SOX-10 expression was 90.8% (95% CI: 75.9% to 105.8%). Statistical heterogeneity was absent: I² = 0% (P = 0.854), indicating highly consistent findings across studies. Pooled proportions represent random-effects meta-analytic estimates and may differ from crude proportions calculated from aggregated case numbers

**Supplementary Fig. 4e** The pooled proportion of SOX-10 positive cases in intraductal carcinoma – apocrine type was assessed across 2 studies, comprising a total of 6 tumors with immunohistochemistry results available. No SOX-10 positive tumors were reported (0 out of 6 cases). The estimated pooled proportion of SOX-10 expression was 10.3% (95% CI: –10.4% to 31.1%). Statistical heterogeneity was absent: I² = 0% (P = 0.610), indicating consistent findings across studies. Pooled proportions represent random-effects meta-analytic estimates and may differ from crude proportions calculated from aggregated case numbers

**Supplementary Fig. 4f** The pooled proportion of SOX-10 positive cases in microsecretory adenocarcinoma was evaluated across 2 studies, comprising a total of 16 tumors with immunohistochemistry results available. SOX-10 positivity was confirmed in all 16 cases. The estimated pooled proportion of SOX-10 expression was 96.1% (95% CI: 87.2% to 105.0%). Statistical heterogeneity was absent: I² = 0% (P = 0.545), indicating highly consistent findings across studies. Pooled proportions represent random-effects meta-analytic estimates and may differ from crude proportions calculated from aggregated case numbers

**Supplementary Fig. 4g** The pooled proportion of SOX-10 positive cases in sialadenoma papilliferum (SP) (all subtypes combined) was evaluated across 2 studies, comprising a total of 20 tumors with immunohistochemistry results available. SOX-10 positivity was detected in 10 out of 20 cases. The estimated pooled proportion of SOX-10 expression was 50.0% (95% CI: 0.0% to 100.0%). No heterogeneity was observed: I² = 0% (P = 1.000), indicating consistent findings across studies
